# Prevalence and predictors of primary and secondary infertility in Namibia: A retrospective analysis of clinical records

**DOI:** 10.4102/curationis.v49i1.2843

**Published:** 2026-09-07

**Authors:** Carolina D. Teixeira, Emanuel Magesa, Sarah Mlambo, Lamech Mwapagha

**Affiliations:** 1 Faculty of Health Sciences, Namibian University of Science and Technology, Windhoek, Namibia; 2 School of Nursing, Faculty of Health, International University of Management, Windhoek, Namibia; 3 School of Public Health, Faculty of Health Sciences, Triumphant College, Windhoek, Namibia; 4 School of Nursing, Faculty of Health, Welwitchia University, Windhoek, Namibia; 5 Department of Biology, Chemistry and Physics, Faculty of Health Natural Resources, Namibia University of Science and Technology, Windhoek, Namibia

**Keywords:** infertility, prevalence, predictors, primary, secondary, patients

## Abstract

**Background:**

Infertility is a public health concern affecting many individuals globally. It is defined as the inability to achieve a pregnancy after 12 months or more of uninterrupted and unprotected sexual intercourse and affects both males and females. Primary infertility occurs when conception has never been achieved, while secondary infertility occurs after at least one previous pregnancy has been achieved.

**Objectives:**

This study analysed the prevalence and predictors of infertility in Namibia. The research was conducted at four selected public hospitals categorised as central, regional and district.

**Method:**

A retrospective and analytical study reviewed 577 hospital records of patients diagnosed with infertility. A structured checklist was developed to extract relevant data, analysed using SPSS version 30 to determine the prevalence and identify predictors of male and female infertility from 2021 to 2023. Ethical approval and permission to access hospital records were obtained from the Namibian Ministry of Health and Social Services and participating hospitals.

**Results:**

Of the 577 patient records, secondary infertility was more prevalent (53.6%) than primary infertility (46.4%). Women constituted 79% of cases, with infertility mainly attributed to hormonal imbalances, tubal, uterine factors and ovulation disorders, while male infertility was largely linked to sperm anomalies. One-way analysis of variance indicated significant differences across age, sex, marital status, infertility type, lifestyle and reproductive history (*p* < 0.05). Multiple regression identified the number of children, sex, age, group, marital status, lifestyle and medication use as significant predictors. Decision tree analysis confirmed parity as the strongest classifier.

**Conclusion:**

Secondary infertility was more prevalent, especially among women, with notable variation across sociodemographic and clinical factors.

**Contribution:**

The findings contribute to the literature on infertility predictors and prevalence in Namibia. This evidence will be used to structure strategies to support individuals affected by infertility.

## Introduction

The World Health Organization (WHO) defines infertility as a disease of the male or female reproductive system, characterised by the failure to achieve pregnancy after 12 months or more of regular unprotected sexual intercourse (WHO 2023). One in every six people in the world has experienced infertility in their lifetime (Liang et al. 2025). Infertility can be classified as primary infertility, which occurs when a person has never achieved pregnancy, and secondary infertility, which occurs when at least one previous pregnancy has been achieved (Santoro & Polotsky 2025).

The prevalence rates of infertility differ by region, with the highest lifetime prevalence reported in the Western Pacific Region at 23.2%, while the Eastern Mediterranean Region has the lowest at 10.7% (Feng et al. 2025). The African Region recorded the highest period prevalence at 16.4%, whereas the Eastern Mediterranean Region had the lowest at 10.0% (Feng et al. 2025). Remarkably, infertility prevalence remains consistent across countries, regardless of income level, with a lifetime prevalence of 17.8% in high-income countries compared to 16.5% in low- and middle-income countries (Liang et al. 2025). This indicates that a person’s poverty status does not determine infertility.

A systematic review found that in Africa, primary infertility prevalence was 49.9%, with North Africa showing the highest rate (70.5%) and East Africa the lowest (30.7%). Secondary infertility was prevalent across regions, particularly in East Africa (69.3%) (Feng et al. 2025).

Specific data for Namibia are limited. However, a study by Lucas and colleagues found that among 178 patients attending the Windhoek fertility clinic, 53.9% had primary infertility and 46.1% secondary infertility, with most participants aged 30–39 years (Lucas et al. 2021).

Infertility is not only a medical condition but also presents a significant psychosocial challenge affecting millions of individuals and couples worldwide, which can considerably impact the quality of life for those affected (Braverman et al. 2024).

Couples and individuals who are unable to conceive often face psychological, emotional and social challenges, such as feelings of grief, loss, anxiety and depression, which are frequently exacerbated by societal pressures and cultural expectations, particularly in societies where childbearing is closely linked to personal worth and social status (Ekpor et al. 2025; Kuug, Siddiwe & Sihaam 2025; Peterson et al. 2025; Ribeiro Neto et al. 2025).

Psychosocial interventions address these emotional and social difficulties by providing coping strategies and emotional support. These interventions effectively reduce anxiety, depression and stress, while also improving the overall quality of life for individuals affected by infertility (Rajamoorthy et al. 2025; Salarfard et al. 2025).

Several factors, categorised into different groups, are known to contribute to infertility globally. These include biological, psychological and unexplained causes, affecting all genders in all countries (Awonuga et al. 2025)

Traditionally, the main causes of infertility were attributed to diseases such as gonorrhoea and other sexually transmitted diseases (STDs) (Thomas 2025; Wei, Huang & Fan 2025). However, the advancements in diagnostic technologies have broadened the understanding of infertility by identifying a wider range of contributing factors, including stress and male-related issues (AlShamlan et al. 2025). Despite these developments, many cases are still classified as unexplained (Jain & Khan 2025). At the same time, the rising incidence of medical conditions such as diabetes, hypertension and hypothyroidism, along with lifestyle-related problems like obesity and substance use, particularly among younger individuals, has also been shown to play a significant role in infertility (AlShamlan et al. 2025).

Various factors are reported to contribute to male infertility, accounting for approximately 50% of cases (WHO 2023). However, these factors remain unidentified in Namibia. These factors may include biological, environmental, lifestyle, psychological and genetic factors (Jain & Khan 2025). Other contributing factors include genital tract infections, cancer treatments, sexual dysfunction and advanced paternal age (Tesarik 2025). Female infertility results from various factors, including biological, lifestyle, environmental, medical and genetic influences (Sakali et al. 2024). These factors may differ by country because of variations in the prevalence of STDs or in the age of the populations studied (Kristensen et al. 2025). Despite this global understanding, data from Namibia remains sparse.

The Health Belief Model (HBM) was used to guide this study in understanding how individual perceptions influence infertility-related behaviours. The model explains that health actions are shaped by people’s beliefs about their susceptibility to a condition, its perceived severity, the benefits of acting and the barriers that may prevent action. In the context of infertility in Namibia, these constructs help to explain why some individuals seek medical care while others delay or rely on traditional medicines. Perceived susceptibility and severity reflect awareness of reproductive health risks and the emotional and social effects of infertility, whereas perceived benefits and barriers influence care seeking and adherence to treatment (Champion & Skinner 2008). By applying the HBM, this study interprets the prevalence and predictors of infertility within the broader framework of individuals’ beliefs and health-seeking behaviours.

This study aims to fill the research gap regarding the predictors and prevalence of infertility in Namibia. Data on the prevalence and predictors are essential, as they will serve as a foundation for understanding the biological causes of infertility. This will inform the development of a psychosocial care model that integrates other datasets on social, psychological and cultural factors to support individuals affected by infertility in Namibia.

### Purpose of the study

The purpose of the study was to analyse the prevalence and the predictors of infertility (male and female) at the four selected public hospitals in Namibia.

## Research methods and design

### Study setting

This retrospective quantitative study was conducted using clinical records from four referral hospitals in three regions of Namibia: Intermediate Hospital, Katutura (Khomas region); Windhoek Central Hospital (Khomas region); Intermediate Hospital Rundu (Kavango East); and Intermediate Hospital Oshakati (Oshana region). These hospitals were purposively selected because they are the four main referral hospitals providing specialised services, including gynaecology and urology services, where infertility cases are diagnosed and managed (Fouché, Strydom & Roestenburg 2021).

### Study design

This study was guided by a positivist research philosophy and employed a quantitative retrospective cross-sectional design. This design was appropriate for determining the prevalence of infertility and identifying factors associated with primary and secondary infertility among patients diagnosed at the selected referral hospitals. A descriptive and analytical approach was used to examine demographic, clinical and lifestyle-related factors associated with infertility.

### Population and sampling

The population in this study consisted of male and female patients aged 18–49 years who were diagnosed with infertility and recorded in the gynaecology and urology clinical registers of the four selected hospitals during the study period from 01 January 2021 to 31 December 2023.

### Inclusion and exclusion criteria

#### Inclusion criteria

Records were included if they met the following criteria:

- Patient diagnosed with infertility and recorded in the gynaecology or urology clinical registers,
- Male or female patients between 18 and 49 years,
- Records were documented during the study period from January 2021 to December 2023,
- Records contained sufficient demographic and clinical information relevant to the study variables.

#### Exclusion criteria

Records were excluded if they:

- Contained incomplete or missing key information required for analysis
- Were a duplicate entry of the same patient

### Sample size and sampling technique

A total population sampling approach was used in this study. All infertility cases recorded in the clinical registers of the selected hospitals during the study period that met the inclusion criteria were included in the analysis. A total of 577 eligible cases were identified from the four hospitals. Since all available records were included, no sampling procedure was applied. This approach ensured comprehensive coverage of all documented infertility cases within the selected facilities during the study period and reduced the risk of sampling bias (Field 2024).

### Instrument and data collection procedure

Data were collected retrospectively from patient records diagnosed with infertility between 01 January 2021 and 31 December 2023. A structured data extraction checklist was developed to guide the collection of information relevant to the study objectives.

The checklist was pretested using a small sample of clinical records from one participating hospital to assess clarity, completeness, and feasibility. These records were not included in the main study. Content validity was ensured by aligning the checklist with the study objectives and variables commonly reported in infertility research. Reliability was strengthened through consistent data extraction procedures and careful verification during data entry and cleaning.

The checklist comprised two sections. Section A captured demographic information, including year of diagnosis, region, age, sex, marital status, employment status, number of children and the hospital attended. Section B focused on clinical and related factors, including type of infertility (primary or secondary), contributing factors (male and female), lifestyle, medical history, laboratory tests performed, current medications and contraceptive use.

Data collection began after approval from the facilities and was conducted between March 2024 and September 2024. Gynaecology and urology registers were accessed with the assistance of the administrative clerks. The registers were organised by financial year and month, which facilitated access.

### Data quality assurance

Several measures were implemented to ensure data quality and accuracy. The structured data. An extraction checklist was developed in accordance with the study objectives and relevant literature. During data collection, each record was carefully reviewed to verify the completeness and consistency of the extracted information. Data were cross-checked during entry to minimise entry errors. After data entry, the dataset was cleaned by identifying missing values, inconsistencies and duplicate records. These procedures ensured the accuracy and reliability of the dataset used for analysis.

### Data analysis

Data were extracted from the clinical registers using a developed structured checklist, checked for errors, cleaned, coded and entered into IBM Statistical Package for the Social Sciences (SPSS) version 30 (IBM Corp, Armonk, NY, United States). A descriptive statistical approach employing frequencies and percentages was utilised to examine the demographic characteristics of the study population and the predictors of infertility in Namibia. Chi-square tests (χ^2^) and analysis of variance (ANOVA) were conducted to assess associations. Binary logistic regression identified predictors of infertility type (primary and secondary). The dependent variable was fertility type, while the independent variables included sex, age group, marital status, lifestyle history, number of children, medical history, factors contributing to infertility, gynaecological and urological history, contraceptive usage history, and other treatments. The descriptive statistics were used to summarise the clinical and demographic characteristics of the 577 infertility cases. Frequencies and percentages were calculated for categorical variables such as age group, gender, marital status, occupation, type and duration of infertility, and identified clinical conditions. This analysis provided an overview of the distribution patterns and emphasised the predominant characteristics among individuals affected by infertility in Namibia. The results are presented in tables and figures to illuminate the proportions and trends observed across different variables.

### Ethical considerations

Ethical approvals for the study were obtained from the Ethics Committee of the Namibia University of Science and Technology (FHNRAS:62/2023), the Ministry of Health and Social Services (Ref: 22/3/1/2), and the selected public healthcare facilities to access the records. Because patient records are regarded as legal documents, strict confidentiality procedures were followed throughout the study. No personal identifiers, such as patient names, identification numbers or contact details, were recorded during data extraction. Data were collected using a structured checklist and stored securely, accessible only to the research team. The study adhered to ethical principles for research involving human data, including confidentiality, privacy and responsible handling of clinical records.

## Results

### Prevalence of infertility

To determine the distribution of infertility types among the study population, descriptive statistical analysis was conducted on records of diagnosed cases. The findings presented in Figure 1 are based on a sample of 577 patient records from the four study sites that were diagnosed with infertility. 309 cases (53.6%) experienced secondary infertility, with a 95% confidence interval (CI) of (49.5–57.7), while 268 participants (46.4%) experienced primary infertility, with a 95% CI of (42.3–50.5).

**FIGURE 1:**
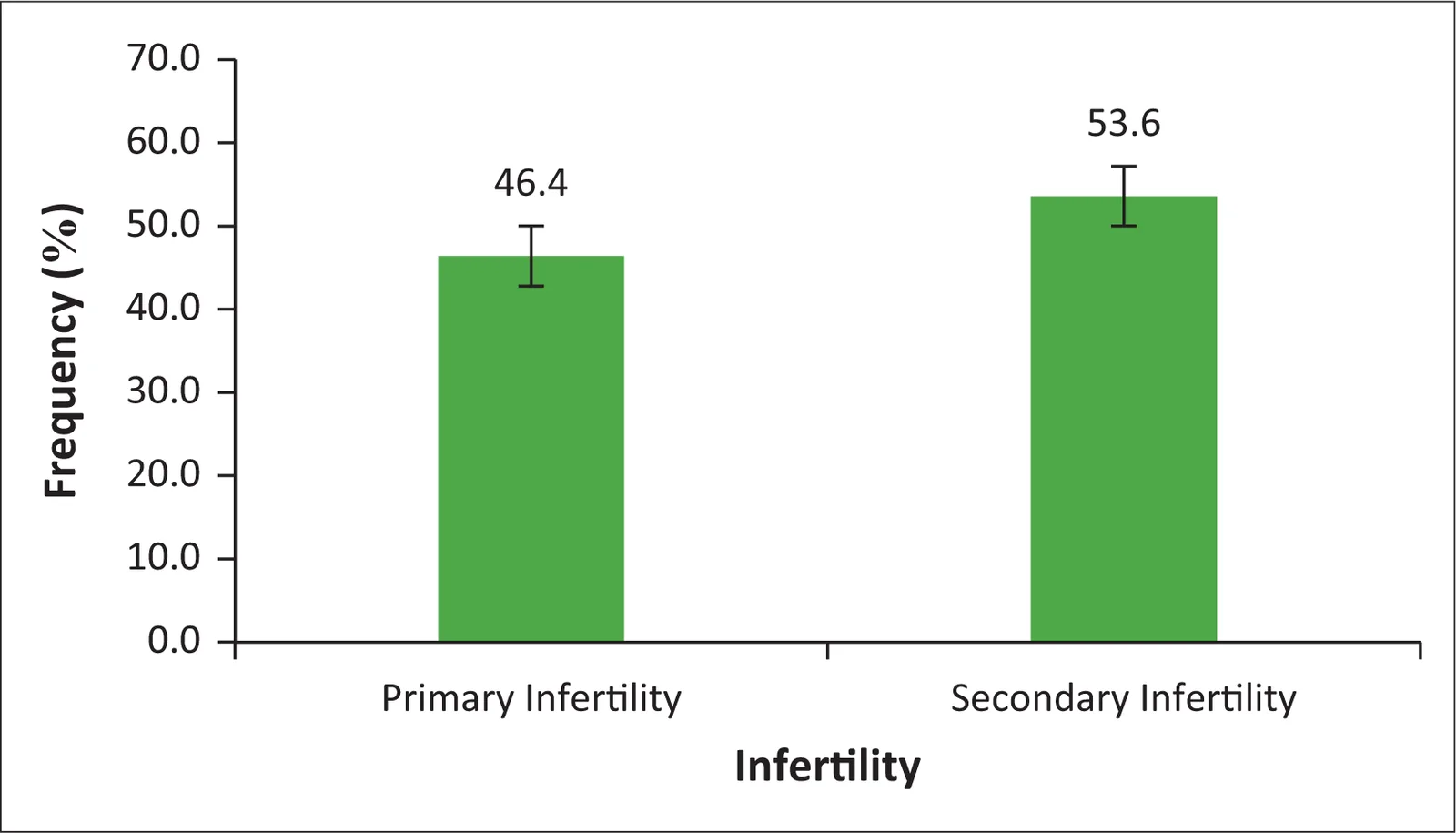
Prevalence of primary and secondary infertility in 577 cases.

### Demographic characteristics and clinical history of the study population

To characterise the study population, descriptive statistics were applied to patient records of individuals diagnosed with infertility. The findings are summarised in Table 1, which presents a sociodemographic profile alongside clinical, lifestyle, reproductive and treatment-related characteristics. The table provides an overview of age distribution, gender composition, marital status, documented lifestyle factors, medical and reproductive histories, medication use, and contraceptive history, offering contextual insight into the background and health profiles of individuals affected by infertility in Namibia during 2021 to 2023.

**TABLE 1:** Social demographic characteristics and clinical history of the study population who were diagnosed with infertility in Namibia from 2021 to 2023 (*N* = 577).

Variables	Sub-variable	Frequency	%
Age group (years)	18–24	34	5.9
	25–29	138	23.9
	30–34	123	21.3
	35–39	147	25.5
	40–44	97	16.8
	45–49	38	6.6
Gender	Male	120	20.8
	Female	457	79.2
Marital status	Single	60	10.4
	Married	205	35.5
	Cohabitating	47	8.1
	Not stated	265	45.9
Lifestyle of participants	Smoking	2	0.3
	Alcohol	22	3.8
	Sedentary lifestyle	1	0.2
	Unspecified	552	95.7
Medical history	Hypertension	17	2.9
	Diabetes	5	0.9
	Asthma	3	0.5
	HIV infection	11	1.9
	Autoimmune polyglandular syndrome	1	0.2
	STIs	66	11.4
	Thyroid problem	2	0.3
	None	149	25.8
	Unspecified	316	54.8
Gynaecological history	Ovarian cyst	14	2.4
	Fibroids	29	5.0
	Hormonal Imbalances	3	0.5
	Amenorrhea	2	0.3
	Breast mass	1	0.2
	Adenomyosis	3	0.5
	Salpingectomy	2	0.3
	Endometriosis	7	1.2
	Irregular bleeding	5	0.9
	None	283	49.0
	Unspecified	111	19.2
	N/A	117	20.3
Urological history	Erectile dysfunction	5	0.9
	Undescendent testes	1	0.2
	Precosse ejaculation	2	0.3
	Varicoceles	3	0.5
	None	2	0.3
	Unspecified	107	18.5
	N/A	457	79.2
Medication or treatment	Folic acid	58	10.1
	TDF/3TC/EFV	5	0.9
	TDF/3TC/DTG	6	1.0
	Amiloride	17	2.9
	Salbutamol	4	0.7
	Calcium Gluconate	1	0.2
	Metformin	5	0.9
	Metformin/Folic acid	4	0.7
	Progesterone	28	4.9
	Ovulation induction treatment	6	1.0
	Folic acid/Multivitamins	7	1.2
	None	298	51.6
	Unspecified	138	23.9
Contraceptive used	Petogen	25	4.3
	Nur-Isterate	26	4.5
	Combined oral contraceptives	5	0.9
	Progestin only pill	6	1.0
	None	299	51.8
	Unspecified	98	17.0
	N/A	118	20.5

STI, sexually transmitted infections; TDF, tenofovir disoproxil fumarate; 3TC, lamivudine; EFV, efavirenz; DTG, dolutegravir.

To determine whether differences existed across groups for selected sociodemographic and clinical variables, a one-way ANOVA was conducted. The results are summarised in Table 2. Significant group differences were observed for most demographic and clinical variables, including age, marital status, infertility type, causes of infertility, lifestyle factors, medical history, gynaecological history, medication use and urological history. Only contraceptive use did not demonstrate a statistically significant difference between groups.

**TABLE 2:** One-way analysis of variance results for group differences across selected variables (*N* = 577).

Variable	Sub-variable	Sum of squares	*df*	Mean square	*F*	*p*-value
Age group category	Between groups	139.453	5	27.891	-	-
	Within groups	904.093	571	1.583	-	-
	Total	1043.546	576	-	17.615	< 0.001
Marital status	Between groups	129.148	5	25.830	-	-
	Within groups	574.613	571	1.006	-	-
	Total	703.761	576	-	25.667	< 0.001
Type of infertility	Between groups	55.293	5	11.059	-	-
	Within groups	88.228	571	0.155	-	-
	Total	143.522	576	-	71.570	< 0.001
The cause of infertility	Between groups	479.154	5	95.831	-	-
	Within groups	10661.886	571	18.672	-	-
	Total	11141.040	576	-	5.132	< 0.001
Lifestyle of the participant	Between groups	10.204	5	2.041	-	-
	Within groups	92.288	571	0.162	-	-
	Total	102.492	576	-	12.627	< 0.001
The medical history of participants	Between groups	275.785	5	55.157	-	-
	Within groups	2372.083	571	4.154	-	-
	Total	2647.868	576	-	13.277	< 0.001
Gynaecological history of participant	Between groups	124.078	5	24.816	-	-
	Within groups	3774.500	571	6.610	-	-
	Total	3898.579	576	-	3.754	< 0.002
The medication that participant taking	Between groups	301.619	5	60.324	-	-
	Within groups	9315.469	571	16.314	-	-
	Total	9617.088	576	-	3.698	< 0.003
Contraceptives used in the past	Between groups	9.656	5	1.931	-	-
	Within groups	1186.375	571	2.078	-	-
	Total	1196.031	576	-	0.930	0.461
Urological history of participant	Between groups	20.214	5	4.043	-	-
	Within groups	312.739	571	0.548	-	-
	Total	332.953	576		7.381	< 0.001
Female or male	Between groups	9.272	5	1.854	-	-
	Within groups	85.771	571	0.150	-	-
	Total	95.043	576	-	12.345	< 0.001

*df*, degrees of freedom.

The multiple linear regression analysis of predictors of the type of infertility is presented in Table 3. The model included some demographic and clinical variables. Number of children (*B* = 0.093, *p* < 0.001) was the strongest predictor of the type of infertility. Other significant predictors included sex, age group, marital status, lifestyle and medication use.

**TABLE 3:** Multiple linear regression analysis of predictors of type of infertility.

Independent variables	*t*-value	*p*-value	95% confidence interval for *B*		Correlations		
			Lower bound	Upper bound	Zero-order	Partial	Part
The medication taking	−2.178	0.030	−0.019	−0.001	−0.079	−0.091	−0.077
Age group	3.206	< 0.001	0.018	0.077	0.219	0.134	0.114
Lifestyle	−2.728	0.007	−0.208	−0.034	−0.039	−0.114	−0.097
Contraceptives used	0.632	0.528	−0.021	0.042	−0.145	0.027	0.022
Gynaecological history	−1.335	0.182	−0.027	0.005	−0.120	−0.056	−0.047
Urological history	−0.233	0.816	−0.075	0.059	0.084	−0.010	−0.008
Female or Male	4.198	< 0.001	0.175	0.482	0.139	0.174	0.149
Marital status	−3.579	< 0.001	−0.102	−0.030	−0.050	−0.149	−0.127
Number of children	12.212	< 0.001	0.078	0.109	0.391	0.457	0.433
The cause of infertility	0.062	0.950	−0.009	0.009	−0.061	0.003	0.002

A decision tree indicating the hierarchical splits of the analysis of 577 infertility cases drawn from the hospital records, classifying primary and secondary infertility (see Figure 2). The initial split was based on the number of children, with further divisions among persons with no children determined by medication use and age group. Proportions of primary and secondary infertility are shown for each branch, illustrating how key variables differentiate between infertility types. This tree shows that patients in the 35–39 age group were more likely to have secondary infertility than primary infertility (63% vs. 36.4%). The use of medication (Metformin, Folic acid, Calcium gluconate, Amiloride and Ovulation induction treatment) was also identified as statistically significant and a predictor of secondary infertility.

**FIGURE 2:**
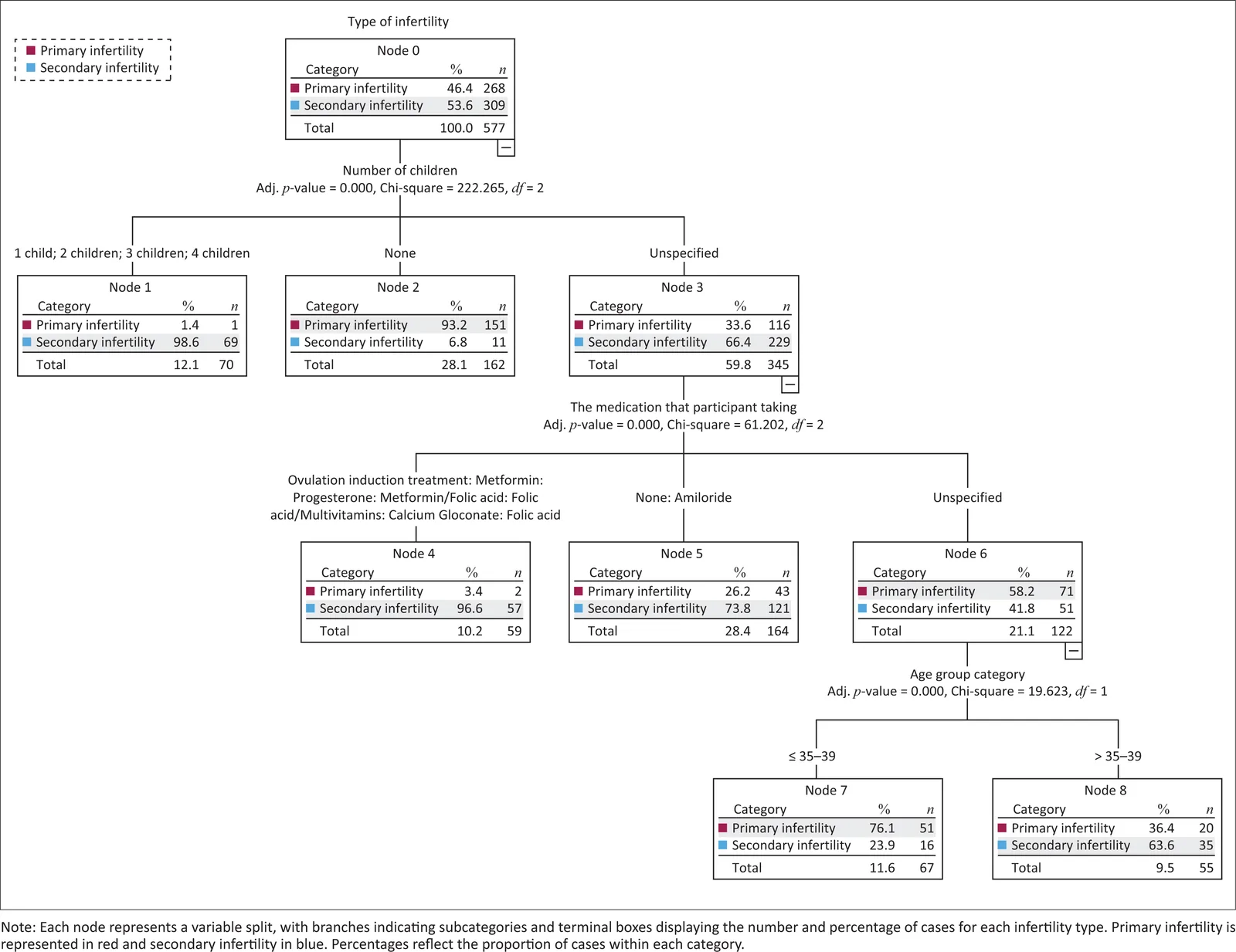
Decision tree classifying primary and secondary infertility based on demographic and clinical predictors.

## Discussion

This study retrospectively analysed the predictors of primary and secondary infertility in 577 patient records from the gynaecological and urology departments of four referral hospitals in Namibia. The findings showed that secondary infertility (53.6%) was slightly more prevalent than primary infertility (46.4%), with primary infertility, with individuals aged 35–39 being the most affected. Marital status and lifestyle factors were identified as significant predictors of infertility (*p* < 0.05). These findings reflect how individuals’ perceptions of risk and health behaviour may influence fertility outcomes, as described in the HBM.

Consistent with our findings, a retrospective study conducted at a referral hospital in Saudi Arabia reported that secondary infertility was more prevalent than primary infertility, accounting for 56.1% and 43.9% of cases, respectively (AlShamlan et al. 2025).

In contrast, a recent study in South Kerala (India) on the prevalence of infertility among married couples reported that 65.8% had primary infertility and 34.2% secondary infertility, reflecting regional variation (Vannya et al. 2025). In low-income countries, the elevated rate of secondary infertility has been linked to sexually transmitted infections, unsterile childbirth conditions and unsafe abortions (Ministry of Health and Social Services [Namibia] 2023).

Similarly, according to the Namibian Ministry of Health and Social Services (MoHSS) report of 2023, Namibia faces a significant burden of sexually transmitted infections, with over 96 000 STI cases recorded at public health facilities over the past 5 years (Esan et al. 2025). Conversely, developed countries have experienced a decline in secondary infertility as a result of reduced fertility preferences, greater access to reproductive technologies and changing social norms (Vannya et al. 2025).

Marital status emerged as a significant predictor (*p* < 0.001), likely reflecting that married individuals are more inclined to attempt conception and seek fertility care. Supporting evidence from a study of 1270 married couples found a 12% prevalence of infertility, with 65.8% reporting primary infertility and 34.2% secondary (Vannya et al. 2025).

Advanced age was significantly associated with infertility in this study, with women aged 35 to 39 representing the group most affected by secondary infertility. This is consistent with the findings by Esan et al., who identified maternal age above 30 years as a strong predictor of female infertility (odds ratio [OR] = 2.88) (Esan et al. 2025). Fertility declines markedly in women after age 35, mainly as a result of declining oocyte quantity and quality (Asherkaci, Baltu & Danfour 2025). Male fertility also declines from age 40–45 onward because of decreased sperm motility and an increased risk of genetic mutations (Choudhary et al. 2025).

The study results revealed that infertility is an emerging reproductive health issue affecting a large portion of the Namibian population. The higher prevalence of secondary infertility was attributed to sexually transmitted infections, lifestyle and advanced age, which contribute to infertility in Namibia. Understanding these patterns is critical for designing interventions that improve fertility outcomes. In the context of the HBM, these factors highlight the role of perceived susceptibility and severity in shaping reproductive health outcomes. Understanding these patterns is important for developing targeted interventions.

The study brings awareness to these predictors of infertility, which will form the basis to strengthen public health strategies that should incorporate lifestyle modification programmes and targeted education on fertility-related risks. Women in rural areas may face additional barriers because of a lack of knowledge on the determinants of infertility, raising the need to enhance the focus on these communities, with a focus on community-based approaches to infertility counselling.

### Limitations of the study

The strength of using a retrospective design employed in this study is the use of existing clinical records, which enabled the inclusion of a relatively large sample within a limited timeframe and with resource requirements compared to prospective approaches. However, reliance on routine hospital records also presents limitations as the data may be compromised by incomplete documentation, variability in reporting and the absence of certain relevant variables.

### Recommendations

Future research should explore the psychosocial dimensions of infertility, particularly stigma and help-seeking behaviours in underserved communities. Male infertility also warrants further investigation. To enhance data quality, an electronic health information system is recommended to reduce missing information and standardise documentation on predictors of infertility across the country.

## Conclusion

This study emphasises that age, marital status, lifestyle factors and the number of children as key predictors of infertility in Namibia. The higher rate of secondary infertility highlights preventable reproductive health issues that need early intervention. Tackling these predictors through comprehensive reproductive health services, public awareness campaigns and policy reforms could markedly reduce the burden of infertility and enhance the reproductive health of Namibians.
